# Individual and organizational factors that affect implementation of evidence-based practices for children with autism in public schools: a cross-sectional observational study

**DOI:** 10.1186/s13012-019-0877-3

**Published:** 2019-03-13

**Authors:** Jill Locke, Gwendolyn M. Lawson, Rinad S. Beidas, Gregory A. Aarons, Ming Xie, Aaron R. Lyon, Aubyn Stahmer, Max Seidman, Lindsay Frederick, Cristine Oh, Christine Spaulding, Shannon Dorsey, David S. Mandell

**Affiliations:** 1 Department of Speech and Hearing Sciences, University of Washington, 1417 NE 42nd St, Seattle, WA 98105 USA; 2 Department of Psychiatry, University of Pennsylvania Perelman School of Medicine, 3535 Market Street, 3rd floor, Philadelphia, PA 19104 USA; 3 Department of Psychiatry, University of California San Diego, San Diego, CA, 9500 Gilman Drive, La Jolla, CA 92093 USA; 4 Department of Psychiatry and Behavioral Sciences, University of Washington, 6200 NE 74th St, Bldg. 29, St. 100, Seattle, WA 98115 USA; 5 Department of Psychiatry and Behavioral Sciences, University of California, Davis, 2825 50th Street, Sacramento, CA 95817 USA; 6 Department of Psychology, University of Washington, Guthrie Hall, Seattle, WA 98195 USA

**Keywords:** Attitudes, Organizational factors, Implementation, Fidelity, Autism, Schools

## Abstract

**Background:**

Children with autism receive most of their intervention services in public schools, but implementation of evidence-based practices (EBPs) for autism varies. Studies suggest that individual (attitudes) and organizational characteristics (implementation leadership and climate) may influence providers’ use of EBPs, but research is relatively limited in this area. This study examined individual and organizational factors associated with implementation of three EBPs—discrete trial training, pivotal response training, and visual schedules—for children with autism in special education classrooms in public elementary schools.

**Methods:**

Participants included 67 autism support teachers and 85 other classroom staff from 52 public elementary schools in the northeastern United States. Participants reported their attitudes toward EBPs (e.g., intuitive appeal, willingness if required, openness, and divergence), implementation leadership and climate of their school, and the frequency with which they deliver each of three EBPs. Linear regression was used to estimate the association of attitudes about EBPs with organizational characteristics and intensity of EBP use. Demographic covariates with a bivariate association with EBP use significant at *p* < .20 were entered into the adjusted models.

**Results:**

There were significant findings for only one EBP, discrete trial training. Teachers who reported higher perceived *divergence* (perceived difference of usual practice with academically developed or research-based practices) between EBPs and current practices used less discrete trial training (*f*^2^ = .18), and teachers who reported higher *appeal* (willingness to adopt EBPs given their intuitive appeal*)* of EBPs used more discrete trial training (*f*^2^ = .22). No organizational factors were significantly associated with implementation with any of the three EBPs.

**Conclusions:**

Attitudes toward EBPs may affect teachers’ decisions to use EBPs; however, implementation leadership and climate did not predict EBP use. Future implementation efforts ought to consider the type of EBP and its fit within the context in terms of the EBP’s similarities to and differences from existing practices and programs in the setting. Implementation strategies that target individual attitudes about EBPs may be warranted in public schools.

## Background

Schools are the primary setting where children with autism, a pervasive developmental disorder, receive intervention services [1–3]. Evidence-based practices (EBPs) are defined as “practices shown by research to have meaningful effects on outcomes” [4], clinical expertise and judgment, and consideration of consumer choice, preference, and culture [5, 6]. EBPs for children with autism [7] such as discrete trial training, pivotal response training, and visual schedules (discussed below) have shown improvements in core symptoms [8–12]. However, fewer than 10% of school-based programs for children with autism are EBPs [13]. Successful implementation of EBPs in schools is challenging because of the complexity and resource-intensive nature of the instructional strategies needed for children with autism [14]. When used, EBPs often are implemented with poor fidelity [4, 15–19], which may decrease the likelihood for improved outcomes.

A number of frameworks have explained why EBPs are or are not implemented in real-world settings in the way they were designed. These frameworks have organized factors affecting EBP implementation across multiple levels (individual, organization, community, system) [20]; however, there is little empirical research on their relative importance and potential value as targets to increase the use of multiple EBPs within the same context. Several studies in community mental health settings have examined the ways individual factors, namely attitudes about EBPs (e.g., intuitive appeal, willingness if required, openness, and divergence), affect EBP adoption and use [21–23]. Attitudes toward EBP adoption and use may hinder or facilitate EBP implementation [24]. Attitudes about EBPs may differ by practitioner demographics [21, 22, 25–28] and type (early intervention vs. mental health providers) [23] and vary within organizations [22]. However, these results are equivocal and stronger research in this area is needed.

Organizational characteristics also provide an important context for understanding successful use of EBPs [27]. Organizational factors, such as implementation leadership (specific leader behaviors that support EBP use) and implementation climate (perceptions on whether EBP use is expected, supported, and rewarded), may play important roles for successful EBP implementation [29, 30]. Recent studies in community mental health settings have shown that both implementation leadership and climate are malleable organizational characteristics [31–33] and may be more proximal to implementation success than other organizational factors such as culture (shared norms and behavioral expectations that guide how work is prioritized and completed in the organization), and climate (staffs’ shared perceptions of the impact of the work environment on their personal well-being), which may take years to change [34–37].

Because one of the goals of implementation research is to understand how the context influences individuals’ implementation in that setting [38, 39], it is important to systematically study individual- and organizational-level constructs in the same context under the conditions in which the setting typically operates. The extent to which attitudes about EBPs and organizational constructs such as implementation leadership and climate relate to the successful use of multiple EBPs, which is a common occurrence in schools, remains an important but unanswered question and can inform the broader implementation science field [40]. Conceptually understanding these factors together will allow for the targeted application of implementation strategies to improve EBP use across multiple levels in complex service systems.

This cross-sectional observational study examines attitudes about EBPs and implementation leadership and climate among special education teachers and classroom staff for children with autism as an illustration of the ways in which individual and organizational factors predict multiple EBP use in public schools. We hypothesized that favorable attitudes about EBPs and stronger implementation leadership and climate will predict intensity of EBP use, whereas individual and organizational factors will separately predict the intensity of EBP use in schools. Multiple EBPs were studied at the same time as that reflects the real-world conditions in which implementation occurs in public school settings. Because simultaneous implementation may result in EBP fatigue and variable usage for school practitioners [42], we hypothesize that attitudes about EBPs and implementation climate and leadership may have differential effects on each EBP.

## Methods/design

### Participants

Participants included 67 Kindergarten-through-third-grade special education teachers and 85 classroom staff (assistants and aides) from 67 classrooms in 52 schools in northeastern United States. Self-contained autism-support classrooms typically have one teacher whose role is to lead all instructional activities and several classroom staff who support instructional activities, which is why there were more classroom staff than teachers. Demographic information for teachers and classroom staff is presented in Table 1.

**Table 1:** Demographic characteristics of participants (*N* = 196)

Characteristic	Teachers *(n = 67)*	Classroom Staff *(n = 85)*
Age in years (*M* (SD))	37.59 (11.26)	43.15 (13.14)
Years experience teaching spec ed. (*M* (SD))	8.57 (7.26)	–
	*N* (%)	*N* (%)
Gender		
Female	65 (97.0)	74 (92.5)
Male	2 (3.0)	6 (7.5)
Race		
White	53 (79.10)	22 (25.9)
Black	11 (16.40)	53 (62.4)
Asian	1 (1.50)	4 (4.7)
American Indian/Alaskan Native	1 (1.50)	1 (1.2)
Not provided	1 (1.50)	2 (2.4)
Educational attainment		
High school	–	18 (21.7)
College	9 (13.4)	29 (34.9)
Some college	–	23 (27.7)
Graduate/professional	56 (83.6)	8 (9.6)
Vocational	–	4 (4.8)
Other	1 (1.50)	1 (1.2)

Seventy-five schools were invited to participate in the study because they had a Kindergarten-third grade autism support classroom. Of these 75 schools, 18 declined to participate and 5 had fewer than three staff working in their autism support classroom or provided substantially missing data (i.e., > 30%) on study measures that prevented data aggregation, resulting in a final sample of 52 schools. Sixty-seven out of 70 teachers (96% response rate) and 85 out of 96 classroom staff (89% response rate) completed data collection.

### Procedure

The university institutional review boards and school district approved the study. The research team met with a school district official to obtain a list of schools with Kindergarten-through-third-grade autism support classrooms. The research team met with the principal at each school to discuss the study activities and obtain a letter of agreement to conduct research. Recruitment materials were distributed to each school, and the research team met with interested participants to inform them about the study and their role as a participant. Once informed consent was obtained, the research team asked participants to complete all study measures. Participants were compensated a $50 check for their time.

Ongoing training was provided to teachers and classroom staff in each EBP (discrete trial training, pivotal response training, visual schedules) for children with autism as part of their standard curricula by a purveyor organization. Didactic group training occurred as part of teachers’ professional development, and monthly in vivo coaching (2 h per/session, once per month) was provided to each classroom in all three EBPs across the school year. Coaches observed teachers’ use of all three EBPs and provided didactic training, modeling, and feedback. For more details on the training, see Pellecchia and colleagues [43]. Discrete trial training is a highly structured, one-on-one instructional strategy that uses massed trials to teach skills where the teacher provides a cue to elicit a response from the child [44]. Pivotal response training uses a naturalistic, play-based approach to address pivotal skills such as motivation and responsivity to the environment to improve language and social skills [46]. Visual schedules involve the use of visual supports to transition students from activity to activity [46]. Although these EBPs are all based on applied behavior analysis [44], the presentation, implementation, and execution of each of these EBPs varies in the classroom [46].

### Measures

#### Dependent variable: intensity of EBP use

Following the methods outlined in Pellecchia and colleagues [19], intensity of EBP use was measured using teacher report for each EBP. Teachers were asked to reflect on their classroom team’s use of each EBP for each student with autism across the previous week. Intensity ratings for discrete trial training and pivotal response training were coded using a Likert scale ranging from “0” to “4” with the following criteria for each score: 0 (less than one time per week), 1 (one time per week), 2 (two to four times per week), 3 (one time per day), and 4 (two times per day). Since visual schedules are used during transitions, teachers used the following scale to rate intensity: 0 (never), 1 (few transitions), 2 (some transitions), 3 (most transitions), 4 (every transition). A research assistant visited each classroom monthly to gather data on intensity of EBP use via teacher and classroom staff report. Teachers’ ratings were averaged across students (range 3–17; *M* = 7.98, SD = 1.92) to determine a classroom score. Intensity of each EBP was averaged across months 5–9 of the school year and the average score was used in all analyses.

#### Independent variables

All measures were adapted in collaboration with the developers for use in the school context. The Evidence-based Practice Attitudes Scale (EBPAS), Implementation Leadership Scale (ILS), and Implementation Climate Scale (ICS), explained below, used in this study were systematically adapted for the school context [40, 41]. All measures were administered toward the beginning of the school year after training in each of the EBPs and prior to assessment of the classrooms’ use of each EBP.

#### Attitudes toward EBPs

Teachers’ and classroom staff’s attitudes about EBP use were measured using the EBPAS, a 15-item measure that assesses four general attitudes toward adoption of EBPs: (1) willingness to adopt EBPs given their intuitive *appeal*; (2) willingness to adopt new practices if *required*; (3) general *openness* toward new or innovative practices; and (4) perceived *divergence* of usual practice with academically developed or research-based practices [25, 41]. The EBPAS subscale reliabilities range from .66 to .91 [21]. The EBPAS has been used with early intervention autism providers [23] and autism support teachers [45]. All subscale scores were used in the analyses.

#### Implementation leadership

The ILS, a 12-item measure with four subscales that assess the degree to which a leader is knowledgeable (deep understanding of EBI and implementation issues), supportive (support staff for EBI adoption/use), proactive (anticipating and addressing implementation challenges), and perseverant (consistent and responsive to challenges) in implementing EBPs, was used [30]. The ILS is a psychometrically validated and reliable instrument (*α* = 0.95–0.98) [30], which has been used in schools [40]. In this study, the leader referent was the principal. Implementation leadership was scored using aggregate individual ratings from teachers and classroom staff. Only the total score was used for analysis given the high correlations among subscales (range: .76–.97) (Table 2).

**Table 2:** Descriptive statistics and correlations for individual and organizational factors

Measure	1	2	3	4	5	6	7	8	9
1. EBPAS—Requirements	–								
2. EBPAS—Appeal	.57**	–							
3. EBPAS—Openness	−.05	.31*	–						
4. EBPAS—Divergence	−.18	−.19	.01	–					
5. ILS—Total Score	.04	−.03	.10	.19	–				
6. ICS—Total Score	.03	.11	.01	.27	.77**	–			
7. Discrete Trial Training	.19	.31*	.06	−.33*	−.24	.30*	–		
8. Pivotal Response Training	−.04	.20	.07	−.17	−.23	−.04.	.43***	–	
9. Visual Schedules	−.03	.11	.11	−.25	−.15	−.10	.16	.34**	–
*M*	3.24	3.11	3.37	.98	2.60	1.97	.71	.55	1.88
SD	.51	1.17	.66	.51	.78	.72	.74	.66	1.64
*N*	61	61	61	61	49	49	67	67	67

***p < .001; **p < .01; *p < .05; *EBPAS* Evidence-based Practice Attitudes Scale, *ILS* Implementation Leadership Scale, *ICS* Implementation Climate Scale

#### Implementation climate

The ICS, an 18-item rating scale that measures employees’ shared perceptions of the policies, practices, procedures, and behaviors that are expected, rewarded, and supported in order to facilitate effective EBP implementation, was used [29]. The ICS has six subscales including (1) focus on EBPs, (2) educational support for EBPs, (3) recognition for EBPs, (4) rewards for EBPs, (5) selection for EBPs, and (6) selection for openness. The ICS is a psychometrically validated and reliable instrument (*α* = 0.81–0.91) [14] and has been successfully used in schools [40]. Implementation climate was scored using aggregate individual ratings from teachers and classroom staff referring to the organization. Only the total score was used for analysis given the high correlations among subscales (range .43–.91).

Concordance between reporters (rwg) was examined for the ILS and ICS. The within-group agreement for the ILS was .68 and the ICC was .28 (range of raters = 3–5); the within-group agreement for the ICS was .78 and the ICC was .32 (range of raters = 3–5). Both the ILS and ICS were averaged across teachers and classrooms staff to create school-level aggregates as exploration of the data supported this [46, 47].

### Data analysis

Study data were managed using Research Electronic Data Capture (REDCap), a secure, web-based application designed to support data capture for research studies [48]. We first examined the distribution of and correlations among variables. We examined unadjusted regression models in which the individual and organizational variables were used individually to predict EBP intensity of each practice. We also examined models that adjusted for demographic covariates (teacher education level and teacher age) that predicted EBP intensity with a *p* value of < .20 [49]. In the adjusted models, teacher education was used as a covariate for predicting discrete trial training intensity and teacher age and teacher education were used as covariates for predicting visual schedules intensity. No demographic factors met criteria to be used as a covariate for predicting pivotal response training intensity.

The a priori sample size calculation was based on 150 participants with *α* = 0.05, which would allow us to detect relatively small associations (Cohen’s *f*^2^ = 0.12). Thirty-eight of the schools had 1 teacher in the sample, 12 schools had 2 teachers in the sample, and 2 schools had 3 teachers in the sample. Because the majority of schools only had one autism support teacher in the sample, we used linear regression to examine associations between attitudes toward EBPs and EBP intensity and each organizational-level factor and EBP intensity. Effect sizes were calculated using Cohen’s ƒ^2^ [50].

## Results

### Intensity of EBP use

The average intensity of discrete trial training use was .72 (*SD* = .74), pivotal response training was .54 (*SD* = 1.20), and visual schedules was 1.88 (*SD* = 1.64). Classrooms on average reported using discrete trial training and pivotal response training less than one time per week and visual schedules only for a “few transitions” throughout the week.

### Individual factors—attitudes

In the unadjusted models, the Divergence subscale on the EBPAS accounted for 10% of the variance in discrete trial training intensity, with lower scores significantly associated with higher intensity (*β* = − .31, *R*^2^ = .10, *p* = .02). In the unadjusted models, the Appeal subscale on the EBPAS accounted for 10% of the variance in discrete trial training intensity, with higher scores significantly associated with higher intensity (β = .31, R^2^ = .10, *p* = .02). No significant associations were found between each individual factor (i.e., Divergence or Appeal) and pivotal response training or visual schedule intensity. The Openness and Requirements subscales on the EBPAS were not significantly associated with intensity of any of the EBPs (see Table 3).

**Table 3:** Unadjusted and adjusted models predicting EBP intensity from individual and organizational factors

Regressions adjusted for covariates						
Variable	DTT intensity		PRT intensity		VS intensity	
	*β*	*R* ^2^	*β*	*R* ^2^	*β*	*R* ^2^
Implementation Leadership Scale (ILS)	−.21	.07	−.23	.05	−.23	.13
Teacher Education	.14		–	–	.00	
Teacher Age	–		–	–	.25	
Implementation Climate Scale (ICS)	−.15	.05	−.04	.00	−.18	.12
Teacher Education	.15		–	–	.01	
Teacher Age	–		–	–	.24	
EBPAS						
Requirements	.24	.09	−.04	.00	−.04	.06
Teacher Education	.22		–	–	−.02	
Teacher Age	–		–	–	.20	
Appeal	.41**	.18	.20	.04	.11	.08
Teacher Education	.28		–	–	.00	
Teacher Age	–		–	–	.20	
Openness	.09	.04	.07	.01	.22	.11
Teacher Education	.17		–	–	−.01	
Teacher Age	–		–	–	.22	
Divergence	−.34**	.15	−.16	.15	−.23	.10
Teacher Education	.23		–	–	.01	
Teacher Age	–		–	–	.21	

**p < .01; *p < .05; *DTT* Discrete Trial Training, *PRT* Pivotal Response Training, *VS* Visual Schedules, *EBPAS* Evidence-based Practice Attitudes Scale

Results were similar in the adjusted models. Lower scores on the Divergence subscale were significantly associated with higher discrete trial training intensity (*β* = − .34, *R*^2^ = .15, *p* = .009, *f*^2^ = .18). Higher scores on the Appeal subscale were significantly associated with higher discrete trial training intensity (*β* = .41, *R*^2^ = .18, *p* = .002, *f*^2^ = .22). The Requirements subscale was not significantly associated with discrete trial training intensity (*β* = .24, *p* = .07).

### Organizational factors—implementation leadership and implementation climate

In the unadjusted models, the ILS total score was not significantly associated with discrete trial training (*β* = − .24, *R*^2^ = .06, *p* = .06), pivotal response training (*β* = − .23, *R*^2^ = .05, *p* = .07), or visual schedules intensity (*β* = − .15, *R*^2^ = .02, *p* = .24). In the adjusted models, the ILS total score was not significantly associated with discrete trial training (*β* = − .21, *R*^2^ = .07, *p* = .10), or pivotal response training (*β* = − .23, *R*^2^ = .05, *p* = .07). In the unadjusted models, the ICS total score was not significantly associated with discrete trial training (*β* = − .17, *R*^2^ = .03, *p* = .17), pivotal response training (*β* = − .04, *R*^2^ = .00, *p* = .74), or visual schedules intensity (*β* = − .10, *R*^2^ = .01, *p* = .41), and the adjusted model also was not significantly associated with discrete trial training (*β* = − .15, *R*^2^ = .05 *p* = .24) (see Table 3).

## Discussion

This study examined individual and organizational factors associated with the simultaneous implementation of three EBPs for elementary-aged children with autism in self-contained classrooms. The results suggest that individual attitudes about EBPs, particularly lower perceived divergence of EBPs with usual practice and greater appeal of EBPs, were associated with one of the EBPs (i.e., discrete trial training). There were no significant associations between individual attitudes about EBPs and use of pivotal response training or visual schedules. Implementation leadership and climate also were not associated with EBP use. These results underscore the importance of considering both individual and organizational factors in the same model and within the implementation context of multiple EBP use.

Individual attitudes and organizational constructs often are examined in isolation despite factors that interact across multiple levels within complex service systems [51]. The results underscore the importance of the intuitive appeal and divergence on use of EBPs, which may suggest the need for a pre-implementation intervention that focuses on altering beliefs and attitudes prior to full-scale EBP training and implementation [52], but organizational factors were not associated with EBP use. Implementation leadership and climate at the broader school level may be too distal to EBP use as principals are too far removed from the classroom to meaningfully influence student outcomes and may not be associated with teacher and classroom staff implementation behavior. It is important to consider the leader referent in various implementation contexts to ensure the most proximal driver of implementation is measured. Future research that examines both individual and organizational factors within the same context is warranted to identify multi-level implementation drivers, particularly in special education [51].

Identifying malleable individual and organizational factors that are associated with implementation of multiple EBPs can inform targeted strategies to improve implementation outcomes and has the potential to mitigate failed implementation efforts, [53, 54], which is common in schools [55]. We found that lower perceived divergence between autism EBPs and usual care practices significantly predicted discrete trial training intensity. This finding is important given the number of EBPs that teachers and classroom staff are expected to use simultaneously, which introduces competing time demands and necessitates prioritization of multiple EBPs [51, 53]. These results highlight the importance of specific intervention characteristics and how they interact with the implementation context. Focusing on a single EBP limits the opportunity to study the fit between different intervention characteristics and implementation contexts [51, 53, 56] and is inconsistent with many settings that implement multiple EBPs to address various mental health conditions [57, 58]. Future research ought to consider multiple EBP use in various implementation settings.

### Limitations

Several limitations should be noted. First, the relatively small sample size precluded our ability to examine mediation or moderation of implementation leadership and climate. Mediation or moderation models may help us understand the nuanced relationships between these constructs and the complications of autism EBP implementation in schools. Second, intensity of EBP use was measured using teacher-report, and there was no measure of other aspects of fidelity such as the quality of intervention delivery, which may be related to practitioner attitudes and implementation leadership and climate. Third, while the implementation leadership and climate scales in this study had a minimum of three raters per school, most raters were teachers and classroom staff from special education settings, which may not represent the majority of the school, the broader perspectives of non-special education employees. Special education classrooms represent a small proportion of the overall school—in our study, 38 schools had one classroom represented; therefore, we were limited in the number of raters per school. Further research is needed to explore how organizational constructs can be more broadly and reliably measured in schools [40]. Fourth, the school district in which these data were gathered is one of the largest school districts in the US and represents a racially/ethnically and socioeconomically diverse population of families and students, which may limit the generalizability to other US school districts. Fifth, multiple analyses were conducted for each set of variables, which may capitalize on chance and lead to increased error. Lastly, while the Domitrovich and colleagues [59] framework guided the study aims, district-level variables that may predict successful implementation and sustainment (e.g., policy, financial constraints) were not measured. This was beyond the scope of the current study but should be considered in future research.

## Conclusion

The results of this study suggest that individual attitudes about EBPs as opposed to organizational factors may be more influential on use of EBPs for children with autism in public schools. Because this study examined the simultaneous use of three EBPs in one context and found significant relationships for one EBP over the others, it is important that future implementation efforts consider the type of EBP and its fit within the context in terms of the EBP’s similarities to and differences from existing practices and programs as EBPs often are not implemented in isolation in schools. The relationship between attitudes about EBPs and implementation outcomes may vary by intervention/EBP characteristics. Because EBP implementation in schools is complicated, it is important to continue to examine the organizational implementation context of schools. However, future research also necessitates exploration of implementation strategies that target individual provider (teachers and classroom staff) attitudes that may improve EBP use for children with autism in public schools.

## Abbreviations

EBP: Evidence-based practice
EBPAS: Evidence-Based Practice Attitude Scale
ICS: Implementation Climate Scale
ILS: Implementation Leadership Scale

## References

[1] Brookman-Frazee L, Baker-Ericzén M, Stahmer A, Mandell D, Haine RA, Hough RL (2009). Involvement of youths with autism spectrum disorders or intellectual disabilities in multiple public service systems. J Ment Health Res Intellect Disabil.

[2] Kang-Yi CD, Locke J, Marcus SC, Hadley TR, Mandell DS (2016). School-based behavioral health service use and expenditures for children with autism and children with other disorders. Psychiatr Serv.

[3] Mandell DS, Cao J, Ittenbach R, Pinto-Martin J (2006). Medicaid expenditures for children with autistic spectrum disorders: 1994 to 1999. J Autism Dev Disord.

[4] Cook BG, Odom SL (2013). Evidence-based practices and implementation science in special education. Except Child.

[5] American Psychological Association. Policy statement on evidence-based practice in psychology. 2005. https://www.apa.org/practice/guidelines/evidence-based-statement. Accessed 4 July 2018.

[6] Institute of Medicine (2001). Crossing the quality chasm: a new health system for the 21st century.

[7] Schwartz IS, Sandall SR, McBride BJ, Boulware G (2004). Project DATA (developmentally appropriate treatment for autism): an inclusive school-based approach to educating young children with autism. Topics Early Child Spec Educ.

[8] Schreibman L (2000). Intensive behavioral/psychoeducational treatments for autism: research needs and future directions. J Autism Dev Disord.

[9] Smith T (2001). Discrete trial training in the treatment of autism. Focus Autism Other Dev Disabl..

[10] Arick J, Loos L, Falco R (2004). The STAR program: strategies for teaching based on autism research.

[11] Koegel RL, Schreibman L, Good AB (1989). How to teach pivotal behaviors to autistic children.

[12] Dettmer S, Simpson RL, Myles BS, Ganz JB (2000). The use of visual supports to facilitate transitions of students with autism. Focus Autism Other Dev Disabl..

[13] Hess KL, Morrier MJ, Hefflin LJ, Ivey ML (2008). Autism Treatment Survey: services received by children with autism spectrum disorders in public school classrooms. J Autism Dev Disord.

[14] Dingfelder HE, Mandell DS (2010). Bridging the research-to-practice gap in autism intervention: an application of diffusion of innovation theory. J Autism Dev Disord.

[15] Mandell DS, Stahmer AC, Shin S, Xie M, Reisinger E, Marcus SC (2013). The role of treatment fidelity on outcomes during a randomized field trial of an autism intervention. Autism..

[16] Locke J, Olsen A, Wideman R, Downey MM, Kretzmann M, Kasari C, Mandell DS (2015). A tangled web: the challenges of implementing an evidence-based social engagement intervention for children with autism in urban public school settings. Behav Ther.

[17] Suhrheinrich J, Stahmer AC, Reed S, Schriebman L, Reisinger E, Mandell D (2013). Implementation challenges in translating pivotal response training into community settings. J Autism Dev Disord.

[18] Stahmer AC, Reed S, Lee E, Reisinger EM, Mandell DS, Connell JE (2015). Training teachers to use evidence-based practices for autism: examining procedural implementation fidelity. Psychol Sch.

[19] Pellecchia M, Connell JE, Beidas RS, Xie M, Marcus SC, Mandell DS (2015). Dismantling the active ingredients of an intervention for children with autism. J Autism Dev Disord.

[20] Tabak RG, Khoong EC, Chambers DA, Brownson RC (2012). Bridging research and practice: models for dissemination and implementation research. Am J Prev Med.

[21] Aarons GA, Hoagwood K, Landsverk J, Glisson C, Kelleher K, Cafri G (2010). Psychometric properties and U.S. National norms of the Evidence-Based Practice Attitude Scale (EBPAS). Psychol Assess.

[22] Smith BD (2013). Substance use treatment counselors’ attitudes toward evidence-based practice: the importance of organizational context. Subst Use Misuse.

[23] Stahmer AC, Aarons GA (2009). Attitudes toward adoption of evidence-based practices: a comparison of autism early intervention providers and children’s mental health providers. Psychol Serv.

[24] Becker-Haimes EM, Okamura KH, Wolk CB, Rubin R, Evans AC, Beidas RS (2017). Predictors of clinician use of exposure therapy in community mental health settings. J Anxiety Disord.

[25] Aarons GA (2004). Mental health provider attitudes toward adoption of evidence-based practice: the Evidence-Based Practice Attitude Scale (EBPAS). Ment Health Serv Res.

[26] Beidas RS, Edmunds J, Ditty M, Watkins J, Walsh L, Marcus S, Kendall P (2014). Are inner context factors related to implementation outcomes in cognitive-behavioral therapy for youth anxiety?. Admin Pol Ment Health.

[27] Beidas RS, Marcus S, Aarons GA, Hoagwood KE, Schoenwald S, Evans AC (2015). Predictors of community therapists’ use of therapy techniques in a large public mental health system. JAMA Pediatr.

[28] Vassos MV, Carroll MF (2016). Assessing attitudes toward evidence-based practices of workers supporting people with disabilities: a validation of the evidence-based practice attitudes scale. Am J Intellect Dev Disabil.

[29] Ehrhart MG, Aarons GA, Farahnak LR (2014). Assessing the organizational context for EBP implementation: the development and validity testing of the implementation climate scale (ICS). Implement Sci.

[30] Aarons GA, Ehrhart MG, Farahnak LR (2014). The implementation leadership scale (ILS): development of a brief measure of unit level implementation leadership. Implement Sci.

[31] Aarons GA, Ehrhart MG, Farahnak LR, Hurlburt MS (2015). Leadership and Organizational Change for Implementation (LOCI): a randomized mixed method pilot study of a leadership and organization development intervention for evidence-based practice implementation. Implement Sci.

[32] Aarons GA, Ehrhart MG, Moullin JC, Torres EM, Green AE (2017). Testing the Leadership and Organizational Change for Implementation (LOCI) intervention in substance abuse treatment: a cluster randomized trial study protocol. Implement Sci.

[33] Aarons GA, Ehrhart MG, Torres EM, Finn NK, Beidas RS (2017). The humble leader: association of discrepancies in leader and follower ratings of implementation leadership with organizational climate in mental health. Psychiatr Serv.

[34] Fixsen DL, Naoom SF, Blasé KA, Friedman R, Wallace F (2005). Implementation research: a synthesis of the literature.

[35] Durlak JA, DuPre EP (2008). Implementation matters: a review of research on the influence of implementation on program outcomes and the factors affecting implementation. Am J Community Psychol.

[36] Forman SG, Olin SS, Hoagwood KE, Crowe M, Saka N (2009). Evidence-based interventions in schools: developers’ views of implementation barriers and facilitators. School Ment Health.

[37] Forman SG, Shapiro ES, Codding RS, Gonzales JE, Reddy LA, Rosenfeld SA, Sanetti LMH, Stoiber KC (2013). Implementation science and school psychology. Sch Psychol Q.

[38] Eccles MP, Mittman BS (2006). Welcome to implementation science. Implement Sci.

[39] Novins DK, Green AE, Legha RK, Aarons GA (2013). Dissemination and implementation of evidence-based practices for child and adolescent mental health: a systematic review. J Am Acad Child Adolesc Psychiatry.

[40] Lyon AR, Cook CR, Brown EC, Locke J, Davis C, Ehrhart M, Aarons GA (2018). Assessing organizational implementation context in the education sector: confirmatory factor analysis of measures of implementation leadership, climate, and citizenship. Implement Sci.

[41] Cook CR, Davis C, Brown EC, Locke J, Ehrart MG, Aarons GA, Larson M, Lyon AR (2018). Confirmatory factor analysis of the evidence-based practice attitudes scale with school-based behavioral health consultants. Implementation Science.

[42] Locke J, Lee K, Cook CR, Frederick, LK, Vazquez-colon C, Ehrhart M, et al. Understanding the organizational implementation context of schools: a qualitative study of school district administrators, principals, and teachers. Sch Ment Health In press; 10.1007/s12310-018-9292-1. 10.1007/s12310-018-9292-1 PMC6824712 31681447

[43] Pellecchia M, Beidas RS, Marcus SC, Fishman J, Kimberly JR, Cannuscio CC, Mandell DS (2016). Study protocol: implementation of a computer-assisted intervention for autism in schools: a hybrid type II cluster randomized effectiveness-implementation trial. Implement Sci.

[44] Arick J, Young H, Falco RA, Loos LM, Krug DA, Gense MH (2003). Designing an outcome study to monitor the progress of students with autism spectrum disorders. Focus Autism Other Dev Disabl.

[45] Locke J, Beidas RS, Marcus S, Stahmer A, Aarons GA, Lyon AR, Mandell DS (2016). A mixed methods study of individual and organizational factors that affect implementation of interventions for children with autism in public schools. Implement Sci.

[46] Brown RD, Hauenstein NM (2005). Interrater agreement reconsidered: an alternative to the rwg indices. Organ Res Methods.

[47] LeBreton JM, Senter JL (2008). Answers to 20 questions about interrater reliability and interrater agreement. Organ Res Methods.

[48] Harris PA, Taylor R, Thielke R, Payne J, Gonzalez N, Conde JG (2009). Research electronic data capture (REDCap)—a metadata-driven methodology and workflow process for providing translational research informatics support. J Biomed Inform.

[49] Bursac Z, Gauss CH, Williams DK, Hosmer DW (2008). Purposeful selection of variables in logistic regression. Source Code Biol Med.

[50] Cohen J (1988). Chapter 2.2: the effect size index-d. Statistical power analysis for the behavioral sciences, 2nd ed.

[51] Stahmer AC, Suhrheinrich J, Schetter PL, Hassrick EM (2018). Exploring multi-level system factors facilitating educator training and implementation of evidence-based practices (EBP): a study protocol. Implement Sci.

[52] Cook CR, Lyon AR, Kubergovic D, Wright DB, Zhang Y (2015). A supportive beliefs intervention to facilitate the implementation of evidence-based practices within a multi-tiered system of supports. School Ment Health..

[53] Rodriguez A, Lau AS, Wright B, Regan J, Brookman-Frazee L (2018). Mixed-method analysis of program leader perspectives on the sustainment of multiple child evidence-based practices in a system-driven implementation. Implement Sci.

[54] Aarons GA, Hurlburt M, Horwitz SM (2011). Advancing a conceptual model of evidence-based practice implementation in public service sectors. Adm Policy Ment Heal Ment Heal Serv Res.

[55] Gottfredson GD, Gottfredson DC (2001). What schools do to prevent problem behavior and promote safe environments. J Educ Psychol Consult.

[56] Green APD, Albanese BJ, Shapiro NM, Aarons GA (2014). The roles of individual and organizational factors in burnout among community-based mental health service providers. Psychol Serv.

[57] Lau AS, Brookman-Frazee L (2015). The 4KEEPS study: identifying predictors of sustainment of multiple practices fiscally mandated in children’s mental health services. Implement Sci.

[58] Beidas RS, Aarons G, Barg F, Evans A, Hadley T, Hoagwood K (2013). Policy to implementation: evidence-based practice in community mental health–study protocol. Implement Sci.

[59] Domitrovich CE, Bradshaw CP, Poduska JM, Hoagwood K, Buckley JA, Olin S (2008). Maximizing the implementation quality of evidence-based preventive interventions in schools: a conceptual framework. Adv Sch Ment Health Promot.

